# Developing measures to capture the true value of primary care

**DOI:** 10.3399/BJGPO.2020.0152

**Published:** 2021-02-17

**Authors:** Tim C olde Hartman, Andrew Bazemore, Rebecca Etz, Ryuki Kassai, Michael Kidd, Robert L Phillips, Martin Roland, Kees van Boven, Chris van Weel, Felicity Goodyear-Smith

**Affiliations:** 1 Radboud Institute of Health Sciences, Department of Primary and Community Care, Radboud University Nijmegen, Nijmegen, The Netherlands; 2 SVP for Research & Policy, American Board of Family Medicine; Co-Director, Center for Professionalism & Value in Healthcare, Lexington, USA; 3 Larry A Green Center for the Advancement of Primary Health Care for the Public Good and Virginia Commonwealth University School of Medicine, Richmond, Virginia, USA; 4 Department of Community and Family Medicine, Fukushima Medical University, Fukushima, Japan; 5 Department of Family and Community Medicine, University of Toronto, Toronto, Canada; 6 Center for Professionalism and Value in Health Care, American Board of Family Medicine Foundation, Washington DC, USA; 7 University of Cambridge, Department of Public Health and Primary Care, Cambridge, UK; 8 Radboud Institute of Health Sciences, Department of Primary and Community Care, Radboud University Nijmegen, Nijmegen, The Netherlands; 9 Radboud Institute of Health Sciences, Department of Primary and Community Care, Radboud University Nijmegen, Nijmegen, The Netherlands; 10 Department of General Practice & Primary Health Care, University of Auckland, Auckland, New Zealand

**Keywords:** measures, universal health coverage, delivery of health care, primary health care, health policy

## Abstract

Primary care (PC) is an essential building block for any high quality healthcare system, and has a particularly positive impact on vulnerable patients. It contributes to the overall performance of health systems, and countries that reorient their health system towards PC are better prepared to achieve universal health coverage. Monitoring the actual performance of PC in health systems is essential health policy to support PC. However, current indicators are often too narrowly defined to account for quality of care in the complex populations with which PC deals. This article reviews a number of conceptual frameworks developed to capture PC values in robust measures and indicators that can inform policy and practice performance. Each have benefits and limitations. Further work is needed to develop meaningful primary health care (PHC) and PC measures to inform strategic action by policymakers and governments for improved overall performance of health systems.

## How this fits in

PC is an essential building block for any high quality healthcare system. Key measures are needed to assess the high value functions of PC and how these translate into reliable, regular indicators of care to inform health policy. A number of conceptual frameworks have been developed to monitor the actual performance of PC in health systems to inform health policy.

## Introduction

Decades of evidence has demonstrated the importance of PC to the health of individuals, populations, and health systems. As an essential building block for any high quality healthcare system,^1^ PC contributes positively to health for all in society, and improves equity in access and outcome.^2–5^ Capable of meeting 80–90% of individuals’ healthcare needs across a lifetime,^6^ PC has a particularly positive impact on vulnerable patients, including those with multiple health problems, people from socially deprived backgrounds, and those with poorly defined illnesses or limited health literacy.^7,8^ A strong PC workforce is associated with lower mortality.^9^ Given ageing populations where multimorbidity becomes the norm, the importance of investing in PC will further increase.^2,10^

PC contributes to the overall performance of health systems through improvements in equity, patient-centredness, access, continuity, coordination, comprehensiveness, quality, and efficiency of care.^6,11,12^ PC is a critical bridge between personal and population health,^13^ and has been the largest source of any care for most people for the past 50 years.^3,14^ Even when health systems with better developed PC are slightly more expensive, as demonstrated in some European contexts,^15^ subsequent reduction of societal costs still present savings.

To implement health policy supportive of PC, it is essential to monitor its actual performance in health systems. And here arises a major problem: health system performance is mainly based on indicators that focus in a disease-specific way on mortality, life expectancy, and management. However, PC is responsible for tending to the large majority of health problems,^16,17^ often not captured in a specific diagnosis, and/or leading to preventive and self-management options. This results in a bias towards secondary care focused on individual diagnoses that are too narrowly defined to account for total quality of care in complex populations with multimorbidity and poor care access.^18,19^

### Relationship between primary care and primary health care

While PHC and PC are often used interchangeably, the World Health Organization (WHO) defines PHC as an approach to health policy and service provision that includes both individual-based care and population-level public health and policy.^20^ Population health includes health promotion, disease prevention, public health, and rehabilitation services. PHC also incorporates wide multi-sectorial functions including community-based social services.

PC involves first contact with patients and their families in their pursuit of health care and health. Individual care involves the health and wellbeing of the whole person and their family throughout all ages and stages of life. Family medicine is the medical discipline providing first contact, and often leadership of a modern PC team. Family doctors can address a patient’s multiple medical issues, and integrate care for individuals and families across the broadest span of PHC.^21^ However, the success of modern family doctors depends on a range of other health professionals in the PC team, including nurses, midwives, pharmacists, mental health workers, and others, depending on setting and context. PC teams with good communication lines between, and close collaboration with, different professionals increase the effectiveness of PC.^22^

In practice, the relationship between PC and PHC is complex. Family medicine may provide both individual and population-level care, such as providing screening and vaccination services for their patient population. PHC as a construct is less associated with individual care, and more on the health of communities and populations, with more explicit incorporation of social, environmental, and public health interventions such as sanitation, water availability and quality, nutrition, and disease epidemic prevention and management. Ideally, PC and PHC work together to address a community’s health needs.

PC services need to be context-dependent with responsive, flexible, and adaptable interventions tailored to specific settings and populations. Individuals or groups with the same health problem may have different needs (control of disease sequelae versus performing daily life tasks); therefore, multiple variables (disease-specific markers versus functional status) are needed to assess outcomes. This makes decisions on what to measure challenging, but the priority measures come back to the essential functions of PC.

Assessing PC using disease-specific measures acts as though the sum of some of its many parts is an indication of its value.^23,24^ This has been referred to as the ‘paradox of primary care’.^19^ While disease-specific measures may suggest that a generalist family doctor might provide a lesser quality of care for a specific disease than a specialist, overall, systems based on PHC can produce healthier populations, use fewer resources, and have less health inequity. Additional measures are required to monitor the impacts of change from strengthening PHC. Recent efforts demonstrate that essential functions of PC can be ‘measured’ and related to important outcomes that matter to patients, clinicians, and insurance companies.^25–27^ Similar core measures of PHC are needed to integrate PC functions, as well as the broader context that PHC offers.

In order for the state of health system quality in low-income and middle-income countries to be assessed and improved*,* the Lancet Global Health Commission on High Quality Health Systems suggests that the core metrics of health system performance should be competent and respectful care, better health, and health system trust, and that other health system components such as finance, management, or organisation are a function of high quality, equitable, efficient care.^28^

A review was conducted of current conceptual frameworks aimed at measuring PC to inform policy and practice performance, based on the authors' collective knowledge of existing tools (Table 1).

**Table 1.** Dimensions and data sources of tools used for measuring the strength of primary care

	**Dimension of care**	**PCAT**	**QOF**	**PHCPI**	**EPCM**	**PCPCM**
Structure or system	Governance				X	
	Economic factors			X	X	
	Workforce				X	
Process or service delivery	Accessibility	X		X	X	X
	Continuity	X		X	X	X
	Coordination	X		X	X	X
	Comprehensiveness	X		X	X	X
	Family or patient-centredness	X				X
	Community orientation	X				X
Outcomes or outputs	Quality	X	X		X	X
	Efficiency			X	X	
	Equity			X	X	
Data sources	Existing datasets		X	X	X	
	Stakeholder survey	X	X		X	X
	Health provider records		X			

PCAT = Primary Care Assessment Tool.^31^ QOF = Quality and Outcomes Framework.^33^ PHCPI = Primary Health Care Performance Initiative.^14^ EPCM = European Primary Care Monitor Framework from Primary Health Care Activity Monitor for Europe (PHAMEU).^48^ PCPCM = Person-Centered Primary Care Measure.^25^

### Primary Care Assessment Tool

The Primary Care Assessment Tool (PCAT), developed in the 1990s, was subsequently extended and validated by Barbara Starfield and colleagues to evaluate service delivery processes across countries.^29–31^ PCAT is designed to measure the extent and quality of PC services from consumer and provider perspectives, and involves a set of three self-report survey instruments (child patient, adult patient, provider). It has been used extensively in some countries, particularly Brazil.^32^ However, each survey can take 40 minutes to complete, which may reduce usability, and the tool focuses largely on processes, not the structure or outcomes of PHC. A further limitation is its US heritage, which transfers poorly to some other countries. However, for developing countries where national datasets are lacking, these self-report surveys may provide a means to assess some components of PHC service delivery.

### Quality and Outcomes Framework

In 2004, the UK NHS introduced the world’s largest healthcare-related pay-for-performance scheme into PC, the Quality and Outcomes Framework (QOF).^33^ Family doctors were paid up to 25% more if they met a complex set of clinical and organisational indicators. The successes of the scheme included an acceleration of previous trends towards systematic management of chronic disease by multidisciplinary teams and widespread introduction of electronic medical records. The QOF did produce improvements in quality of care,^34–36^ but these were modest, against a background of national quality improvement programmes, including national standards for the major chronic diseases, annual appraisal of all doctors, and widespread use of clinical audits to compare practices, sometimes with public release of data. There were reductions in inequities in delivery of care for the major chronic diseases in QOF, with practices in socioeconomically deprived areas catching up with the performance of practices in more affluent areas.^37^ The scheme may have limited increase in emergency admissions for incentivised conditions,^38^ but has not reduced mortality.^39,40^

QOF has almost certainly had some negative consequences, for example, the progressive decline in the ability of patients to see a family doctor of their choice is probably an unintended consequence of a relentless focus by government on incentives for broad and immediate access to care. Similar experience is emerging from the US’s singular attention to open access as a part of Patient-Centered Medical Home transformation. There is also concern that schemes that focus on single-condition guidelines and indicators lead to overtreatment of increasing numbers of older patients, a failure to effectively address multimorbidity in the population, and the potential neglect of aspects of care that are not readily measured and, hence, not included in these schemes. Although initially popular, family doctors have become increasingly disenchanted with the administrative workload of the QOF scheme. In 2017, Scotland dropped QOF entirely in favour of incentives for family doctors to work in quality circles (groups of family doctors working together to improve quality).^41^ However, there has also been concern that removing indicators from the incentive scheme may result in reductions in quality of care.^42^

Overall, the experience with QOF is consistent with evidence from other parts of the world; for example, financial incentives are often less effective than expected and they can have unintended consequences, particularly if the true high value functions are not part of the measure and incentive policy.^43^ Challenges for those introducing incentive schemes include: determining that they genuinely meet the health needs of the population; ensuring they assess and incentivise the most important behaviours; and respecting that population health needs change.

### Primary Health Care Performance Initiative

The World Bank, Bill & Melinda Gates Foundation, and the WHO formed the Primary Health Care Performance Initiative (PHCPI) to strengthen measures, specifically in low- and middle-income countries (LMIC).^14^

The PHCPI conceptual framework looks at system, input, service delivery, output and outcome factors addressing issues related to PHC financing, capacity, performance, and equity, and more recently management of services, including team-based approaches and community engagement.^44^ The indicators used by PHCPI have recently been revised to focus greater attention on those areas expected to have the greatest impact in supporting countries to attain universal health coverage (UHC).^45^ The Vital Signs Profile was designed to provide a comprehensive snapshot of the performance of a country’s PHC system, but is limited by availability of existing data sources. While PHCPI provides a robust and systematic system for measuring PHC performance, there remain gaps.^46,47^ While adequate measures have been identified for topics, such as PHC spending, access, quality, service coverage, and health outcomes, these have been lacking for assessing the components of PHC that impact overall performance and outcomes.

PHCPI has recently developed the PHC Progression Model with a mixed-method assessment tool for more comprehensive and systematic measurement of PHC capacity.^46^ While this tool shows promise in enabling countries to track their progress over time and make cross-country comparisons, the assessment requires investment in time and other resources.

### European Primary Care Monitor Framework

Kringos and colleagues provide a conceptual framework incorporating structural (governance, economic conditions, and workforce development), service delivery processes (accessibility, comprehensiveness, continuity, and coordination), and outcome (quality, efficiency, and equity of care) dimensions of PC.^3^ Their work was developed across the relatively high income European countries, although it included the Eastern European ‘transition’ countries.^48^ They operationalised the ‘European Primary Care Monitor’, with a set of 99 indicators, as weak, medium, or strong PC, enabling overall totals of each dimension to be calculated.^49^

The Primary Health Care Activity Monitor for Europe (PHAMEU) project used these indicators to compare secondary data from 31 European countries sourced from national and international databases and the scientific literature, augmented by national experts who accessed grey literature to fill in the gaps.^48^ They found that countries either had strong PC structures (good financial coverage and resources, and adequate workforce) or few of these structures in place. They found no correlation between performance on the four dimensions of accessibility, comprehensiveness, continuity, and coordination. However, strong PC was associated with better population health, lower rates of unnecessary hospitalisation, and relatively lower socioeconomic disparities. These authors have also found that countries with stronger health structures have overall higher health expenditures.^15^ This may be because they have a greater investment in health overall, not necessarily that PHC is more expensive than hospital-based services. Those systems with comprehensive PC were also associated with slower growth in healthcare spending.

### Person-Centered Primary Care Measure

When conducting patient assessment of PC delivery, it is essential to acknowledge PC’s complexity: it addresses the whole person while integrating experience, prioritising needs, and personalising care. The purpose of measuring is to focus attention on what matters, but the current measures focused on disease-specific processes and clinical outcomes point in the wrong direction. Their selective focus institutionalises fragmentation and enacts a basic misalignment between what is measured and what is valued.^25^ This takes the focus away from what is important to PC, which is why a person seeks care, and what help can be provided.^24^ A conceptual PC framework should include high quality clinical care and interpersonal communication. Measures are needed that are capable of assessing PC’s complexity; for example, in addressing professionalism, aligning with the experience of patients and clinicians, eliminating fragmentation, and including the social and relational aspects known to contribute to its success.^25^

A first requirement is to find language to describe the work PC does, and to define criteria for its assessment. That is the purpose of the newly developed Person-Centered Primary Care Measure (PCPCM). The first phase of development started with a crowdsourced survey of what patients and clinicians felt represented good PC, resulting in 18 identified quality indicator areas.^25^ The second phase occurred at the Starfield Summit III: Meaningful Measures for Primary Care (international conference in Washington DC, October 2017), during which the 18 quality indicator areas were shared with 70 expert stakeholders, including patients, providers, payers, and measure creators, and were reduced to 11 main indicator areas (that is, accessibility, comprehensiveness, continuity, integration, coordination, relationship, advocacy, family context, community context, health promotion, and goal-oriented care). Each main indicator area was captured in a single written item using the language found in the crowdsourcing and conference data.

PCPCM has been shown to have good reliability and construct validity.^25^ Crowdsourced and Starfield III findings support its alignment with clinician-identified aspects of professionalism, and the overlap of quality areas surveyed between patients and clinicians. PCPCM is robust and capable of capturing the essential functions through which PC operates. Further studies regarding the measure’s performance and its potential use globally are underway.

## Conclusion

Strong health systems require government commitment to provision of UHC, alignment of partners and stakeholders to support national strategies and policies, and individuals and communities empowered to enhance their own health and wellbeing. Health systems vary widely across different countries. PC is different from, but essentially overlapping with, PHC. PC’s high value functions are universal, but their translation into services provided are context-specific and depend on population needs, the health professionals available to provide care, population socioeconomic factors, and a political commitment to PHC. Describing and measuring the nature and quality of both PC and PHC services enables comparison between national health systems, the ability to identify best practice, and can inform strategic action by policymakers and governments. The frameworks summarised above are innovative steps towards better evaluation of the foundational aspects of PC and PHC. However, only the systematic application in performance measurement of PC and PHC around the world — in different health systems and socioeconomic and cultural settings — will clarify their true strengths and limitations.

Measurement needs to be meaningful to those who deliver care. They need a sense of ownership of the processes of change, and the indicators needed to guide this. Key measures need to address the high-value functions of PC and how these translate into reliable, regular indicators of care; the patient experience of PHC; the weight to be given to clinical measures as indicators of practice and health systems, to reduce or avoid perverse effects; and how the international PC community can best work with WHO, the Bill & Melinda Gates Foundation, and others to support person-centred and population-centred care through PHC. Meaningful PHC and PC measurement is essential to improve overall performance of health systems in the move towards achieving UHC.
